# Independent Neuronal Origin of Seizures and Behavioral Comorbidities in an Animal Model of a Severe Childhood Genetic Epileptic Encephalopathy

**DOI:** 10.1371/journal.pgen.1005347

**Published:** 2015-06-30

**Authors:** Samuel K. Asinof, Stacey J. Sukoff Rizzo, Alexandra R. Buckley, Barbara J. Beyer, Verity A. Letts, Wayne N. Frankel, Rebecca M. Boumil

**Affiliations:** 1 The Jackson Laboratory, Bar Harbor, Maine, United States of America; 2 Biomedical Sciences Graduate Program, University of California, San Diego, San Diego, California, United States of America; Stanford University School of Medicine, UNITED STATES

## Abstract

The childhood epileptic encephalopathies (EE’s) are seizure disorders that broadly impact development including cognitive, sensory and motor progress with severe consequences and comorbidities. Recently, mutations in *DNM1* (dynamin 1) have been implicated in two EE syndromes, Lennox-Gastaut Syndrome and Infantile Spasms. *Dnm1* encodes dynamin 1, a large multimeric GTPase necessary for activity-dependent membrane recycling in neurons, including synaptic vesicle endocytosis. *Dnm1^Ftfl^* or “fitful” mice carry a spontaneous mutation in the mouse ortholog of *DNM1* and recapitulate many of the disease features associated with human *DNM1* patients, providing a relevant disease model of human EE’s. In order to examine the cellular etiology of seizures and behavioral and neurological comorbidities, we engineered a conditional *Dnm1^Ftfl^* mouse model of *DNM1* EE. Observations of *Dnm1* ^Ftfl/flox^ mice in combination with various neuronal subpopulation specific cre strains demonstrate unique seizure phenotypes and clear separation of major neurobehavioral comorbidities from severe seizures associated with the germline model. This demonstration of pleiotropy suggests that treating seizures *per se* may not prevent severe comorbidity observed in EE associated with dynamin-1 mutations, and is likely to have implications for other genetic forms of EE.

## Author Summary

Childhood epilepsy syndromes, such as the early epileptic encephalopathies (EE’s) encompass seizure disorders that manifest early and negatively impact or completely block developmental progression. Recently, mutations in *DNM1* (dynamin 1) have been implicated in two EE syndromes, Lennox-Gastaut Syndrome and Infantile Spasms. Dynamin 1 is a large multimeric protein that is critical for electro-chemical communication between neurons. To understand the relationship between severe seizures and the cognitive and behavioral developmental outcomes in DNM1 patients, we focus on “fitful” mice that carry a mutation in the dynamin 1 gene. Fitful mice have an EE disorder that is highly reminiscent of the documented human patients. Here, we describe genetic manipulations in the mice that allow us to determine that the seizure activity has independent cellular origins from the developmental and behavioral consequences. This separation confirms that the seizures do not cause the severe developmental delay and abnormal behaviors in this animal model and further suggests that any treatments aimed at controlling the seizures *per se* may not be effective for some of the most acute neurobehavioral symptoms in these patients.

## Introduction

Epileptic encephalopathies (EE’s) encompass a group of seizure disorders that impact overall development including cognitive, sensory and motor progress with severe developmental consequences and comorbidities. Recently, *de novo* missense mutations in *DNM1* have been implicated in two EE’s, Lennox-Gastaut Syndrome and Infantile Spasms. Dynamin 1 (*DNM1*) is a large GTPase necessary for synaptic vesicle recycling and endocytosis at the presynapse.

Currently, at least eight patients have been documented with mutations in *DNM1* [1–3]. This number is likely to grow based on the increasing efficacy of genetic diagnosis of EE by genome sequencing. Affected children suffer from infantile spasms that progress to seizures, global developmental delay, profound intellectual disability, lack of speech and general hypotonia. All patients carry *de novo* heterozygous missense variants which are most likely dominant negative based on *in vitro* studies [4]. Studies of “fitful” mice, which carry a spontaneous missense mutation in the *Dnm1* gene, have demonstrated a dominant negative effect of the mutant protein *in vitro* and the mice display seizures and neurosensory defects [5].

**Fig 1:**
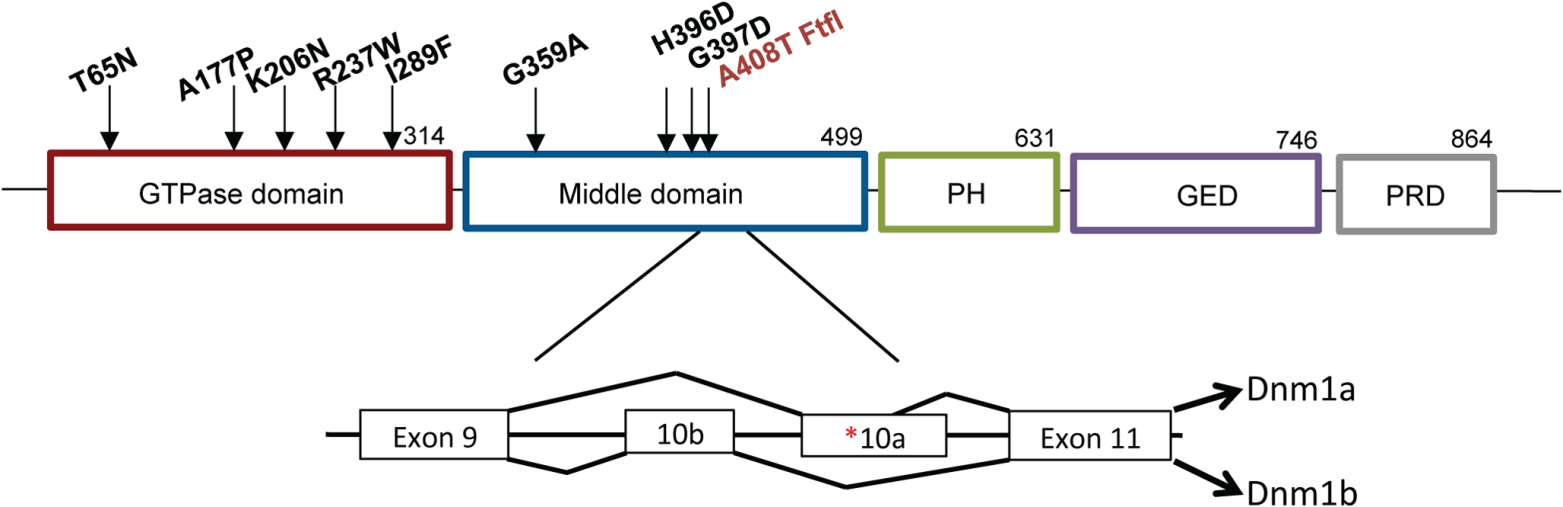
Structure of DNM1. Schematic of DNM1 protein showing the domain locations of eight human variants [1–3] and the fitful mutation ([5]; in red) within an alternatively spliced exon in the middle domain. The exon structure shows the location of the fitful mutation in the alternatively spliced exon 10a. Dnm1a, containing exon 10a, and Dnm1b, containing exon10b, can functionally compensate for each other.

The fitful mouse model recapitulates many of the features of the *DNM1* EE syndrome. The homozygous mice have early onset seizures, ataxia and general decline in overall activity and health, succumbing to a seizure and demise before four weeks of age. The fitful mutation resides in an alternatively spliced exon and wildtype *Dnm1* is produced from the alternate exon (Fig 1). Thus, the homozygous mice are analogous to the heterozygous patients with respect to having both wildtype and mutant dynamin 1 expressed. While a *Dnm1* knockout mouse has been studied [6], neither the heterozygous nor homozygous mice have seizures and therefore do not recapitulate the syndrome observed in humans, further suggesting that both the EE-causing human *DNM1* variants and fitful are gain-of-function mutations.

Homozygous fitful mice (*Dnm1* ^*Ftfl/Ftfl*^) are severely affected: they exhibit a progressive ataxic movement disorder, typically lethal tonic-clonic seizures and die before four weeks of age [5]. Fitful heterozygotes (*Dnm1* ^*Ftfl/+*^) are less severe with no movement disorder and non-lethal tonic-clonic seizures that have an onset at about two to three months of age [5]. Heterozygous *Dnm1* knockout mice (*Dnm1* ^*null/+*^) do not have any overt phenotype, whereas homozygous knockout mice exhibit perinatal lethality with a general failure to thrive, but neither genotype has seizures [6]. Compound heterozygous mice expressing the fitful mutation in combination with the null allele have been described previously (*Dnm1* ^*Ftfl/null*^; [5]). The onset and appearance of lethal seizures is very similar between fitful homozygotes and compound heterozygotes, but their locomotor deficit manifests in a slightly different phenotype; the compound heterozygotes are not ataxic, although they exhibit a slight tremor and hunched appearance that increases continually in severity until death. See Table 1 for a summary of the dynamin 1 alleles referred to in this study.

**Table 1:** Dynamin 1 mutant genotypes referenced in this study.

	Fitful heterozygote	Fitful homozygote	Dnm1 KO heterozygote	Dnm1 KO homozygote	Fitful conditional	Fitful compound heterozygote
*Dnm1* genotype	*Dnm1* ^*Ftfl/+*^;[5]	*Dnm1* ^*Ftfl/Ftfl*^;[5]	*Dnm1* ^*null/+*^;[6]	*Dnm1* ^*null/null*^;[6]	*Dnm1* ^*Ftfl/flox*^;[5,7]	*Dnm1* ^*Ftfl/null*^;[5,6]
Seizures	yes	yes	no	no	yes	yes
*Age at seizure onset*	2–3 months	~14 days	(N.A.)	(N.A.)	2–3 months	~14 days
*Age at 100% lethality*	(N.A.)	~23 days (lethal seizure or general decline)	(N.A.)	7–8 days old (decline)	(N.A.)	~23 days (lethal seizure or general decline)
*Observable abnormal behavior*	none	Hunched posture; ataxic gait, gait worsens with seizure onset and frequency	none	(N.A.)	none	Hunched posture; fine tremor; gait worsens with seizure onset and frequency

N.A., not applicable.

The first row indicates the name of the mouse line referred to in the study. The *Dnm1* genotype indicates the composition of the two dynamin-1 alleles for that mouse line and is followed by the reference which created or characterized the mouse or allele. The table describes the seizure and behavior phenotypes for the particular mouse line/dynamin allele combination.

In a given genetic disease it can be very difficult to distinguish syndromic comorbidities due to pleiotropy from those resulting from damage attributable to the seizures themselves–both are plausible (reviewed in [8,9]). In order to make the distinction for DNM1 epileptic encephalopathy, we engineered a conditional *Dnm1* ^*Ftfl*^ mouse model of *DNM1* EE. Observations of *Dnm1* ^Ftfl*/*flox^ mice in combination with various neuronal subpopulation specific cre strains demonstrate unique seizure phenotypes and establish the separation of comorbidities from seizures.

## Results

### Generation of a conditional *Dnm1* ^*Ftfl/null*^ EE activating mouse line

**Fig 2:**
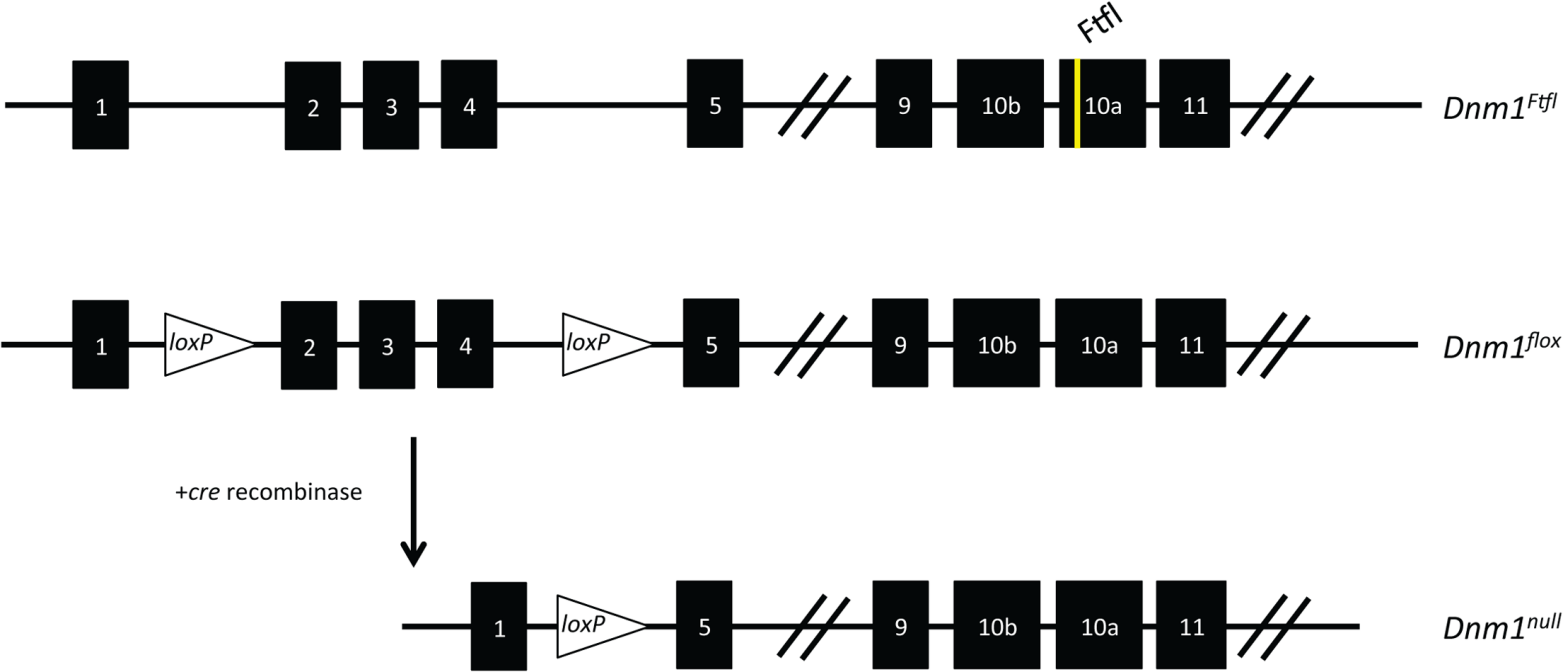
Schematic of the *Dnm1* transcript and the conditional EE genotype strategy. (Top) The *Dnm1* ^*Ftfl*^ allele is shown with the mutation in alternatively spliced exon 10a. (Middle) The *Dnm1* ^*flox*^ allele is shown with location of the loxP sites flanking exons 2–4 [7]. (Bottom) After cre recombinase activity, exons 2–4 are deleted resulting in a frameshift and the *Dnm1* ^*null*^ allele. Thus, the genotypes in the present studies are compound heterozygous, *Dnm1* ^*Ftfl/null*^, where the null (deleted allele) is present only in cells that express cre recombinase.

In order to study the fitful mice more closely, we made a less severe conditional model by combining the dominant-negative *Ftfl* allele (*Dnm1* ^*Ftfl/+*^) with a *Dnm1* floxed allele (*Dnm1* ^*flox/+*^; Fig 2, [7]). The floxed allele has loxP sites flanking exons 2–4 of *Dnm1* and upon recombination using cre recombinase technology the exons are deleted resulting in a frameshift and the *Dnm1* null allele (Fig 2). Mice carrying the *Dnm1* ^*Ftfl*^ and the intact floxed allele (*Dnm1* ^*Ftfl/flox*^) are phenotypically identical to fitful heterozygous mice (Table 1). When a ubiquitous cre recombinase is used to delete the floxed allele, the resulting mice are referred to as *Dnm1* ^*Ftfl/null*^ and phenotypically resemble the compound heterozygous *Dnm1* ^*Ftfl/null*^ germline mice (Table 1). Further and importantly, this allelic combination provides a genetically manipulatable system in which the severe *Dnm1* ^*Ftfl/Ftfl*^ homozygous or compound heterozygous *Dnm1* ^*Ftfl/null*^ genotype can be recapitulated in specific neuronal populations using cell specific cre recombinase expression (Fig 2). Thus the contribution of particular neuronal populations to the *Dnm1* fitful severe phenotype can be assessed. It is worth noting that cells that do not express the cre remain *Dnm1* ^*Ftfl/flox*^ heterozygous, the milder fitful genotype which exhibits a non-lethal seizure disorder with late onset.

**Fig 3:**
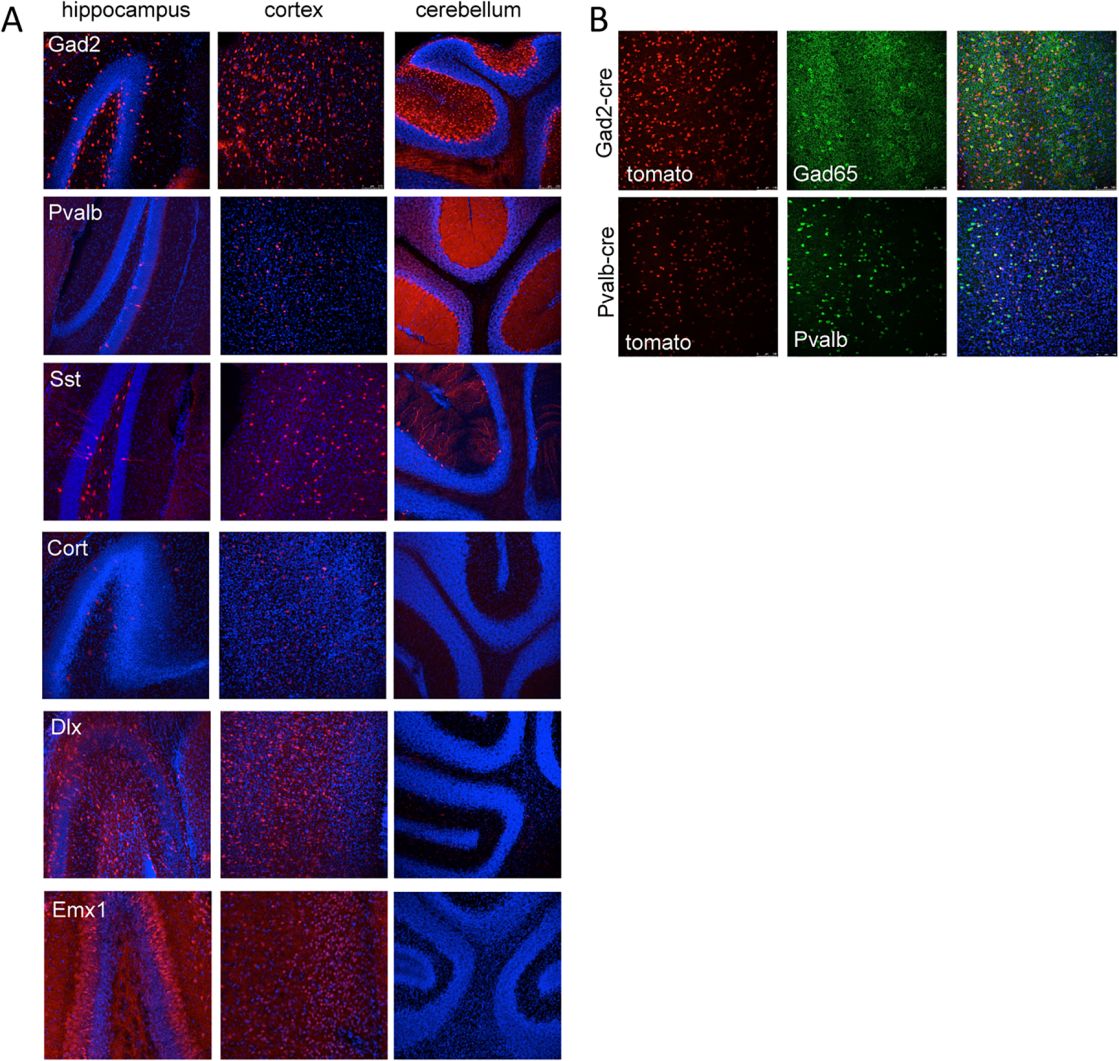
Tomato reporter expression in brain sections from gene specific cre strains. (A) Vibratome sections from the hippocampus, cortex and cerebellum of *Gad2*-cre, *Pvalb*-cre, *Sst*-cre, *Cort*-cre, *Dlx5/6*-cre and *Emx1*-cre mice crossed to mice carrying the conditional red fluorescent protein variant tdTomato. tdTomato is expressed only in cells expressing the cre (red), nuclei are counterstained with DAPI (blue). (B) Vibratome sections from the cortex of *Gad2*-cre (top images) or *Pvalb*-cre (bottom images) expressing animals crossed with the tomato reporter and counterstained with Gad65 antibody (top) or parvalbumin antibody (bottom) demonstrating the overlap of the cre expression with inhibitory neurons (Gad65 or Pvalb).

We used several different cre recombinase-expressing mouse lines to determine the outcome of dominant negative fitful expression in specific neuronal cell populations. Table 2 details the cre mouse lines used for this study and the timing and expression in the brain according to published data. Further, to confirm the timing and expression of these lines in our hands, we crossed the various cre lines to a reporter line with a floxed-stop red fluorescent protein variant tdTomato [10]. Fig 3 shows representative sections of hippocampus, cortex and cerebellum from cre line mice crossed to tomato reporter lines. As *Gad2*-cre is expressed widely in inhibitory neurons [11], these mice have the most extensive reporter expression of the inhibitory lines in the tissues examined (Fig 3A and 3B). In the *Pvalb*-cre mice, which express cre in parvalbumin positive inhibitory neurons, there is sparse hippocampal expression in the dentate gyrus region with greater expression in the cortex and high expression in the cerebellum (Fig 3A and 3B). The *Sst*-cre is expressed in somatostatin positive inhibitory cells and the mice show the expected patterns in the dentate gyrus within cells residing in the hilus and projecting onto granule cells, with high expression in the cortex and discrete cells, such as Purkinje cells, in the cerebellum as well (Fig 3A). *Cort*-cre is expressed in cortistatin positive inhibitory neurons and the mice have expression in the cortex and hippocampus, but not the cerebellum (Fig 3A). *Dlx5/6*-cre expresses in inhibitory neurons of the forebrain and we see very little to no expression in the cerebellum (Fig 3A) or brainstem. *Emx1*-cre is highly expressed in the majority of excitatory neurons in the hippocampus and cortex, but not outside the forebrain (Fig 3A; [12]).

**Table 2:** Expression patterns associated with cre recombinase mouse lines used in this study.

Cre line; ref	*Gad2*-cre [11]	*Pvalb*-cre [13]	*Sst*-cre [11]	*Cort-cre* [11]	*Dlx*-cre [14]	*Emx1*-cre [12]
Expression pattern						
	Olf bulb++++	Olf bulb -	Olf bulb ++	Olf bulb -	Olf bulb +++	Olf bulb ++
	Cortex ++++	Cortex ++++	Cortex ++	Cortex ++	Cortex +++	Cortex ++++
	Hippo ++++	Hippo ++++	Hippo ++	Hippo ++	Hippo ++	Hippo ++++
	Amyg ++++	Amyg ++++	Amyg ++	Amyg -	Amyg ++	Amyg +
	Cere ++++	Cere ++++	Cere +	Cere -	Cere -	Cere -
	Striatum ++++	Striatum ++++	Striatum ++	Striatum -	Striatum ++	Striatum -
	Hypothal ++++	Hypothal ++++	Hypothal ++	Hypothal -	Hypothal -	Hypothal -
	Brain St ++++	Brain St ++++	Brain St ++	Brain St -	Brain St -	Brain St -
Timing						
	embryonic	hindbrain, embryonic	embryonic	cortex, embryonic	embryonic	embryonic
		forebrain, >P7		hippocampus, >P7		
Overlapping expression						
	Overlap with Pvalb, Sst, Cort, Dlx	Partial overlap with Cort	Partial overlap with Cort	Partial overlap with SST, Pvalb	Partial overlap with Pvalb, Sst, Cort	

The first 5 cre lines are inhibitory neuron targeted and the *Emx1* line is excitatory neuron expressed. References detailing the creation and characterization of the lines follow each cre line name. The + and–symbols are used to represent relative expression ranging from high (++++) to none observed (-). Timing indicates when the cre is first observed to express and where if isolated expression is observed initially.

The extent of deletion of the floxed allele was assayed at the protein level. The *Dnm1* ^*Ftfl*^ allele is expressed in the entire brain of the *Dnm1* ^*Ftfl/flox*^ animals, therefore only a decrease in *Dnm1* abundance, not a complete deletion, can be detected. We detect a decrease approaching 50% in overall Dnm1 abundance in protein from cortical lysates of *Emx1*-cre;*Dnm1* ^*Ftfl/flox*^ animals compared to wildtype littermates (S1 Fig), indicating that the floxed allele is being deleted from a majority of cells.

### *Dnm1* ^*Ftfl/null*^ EE phenotype in inhibitory neurons leads to hyperexcitability and premature death

**Fig 4:**
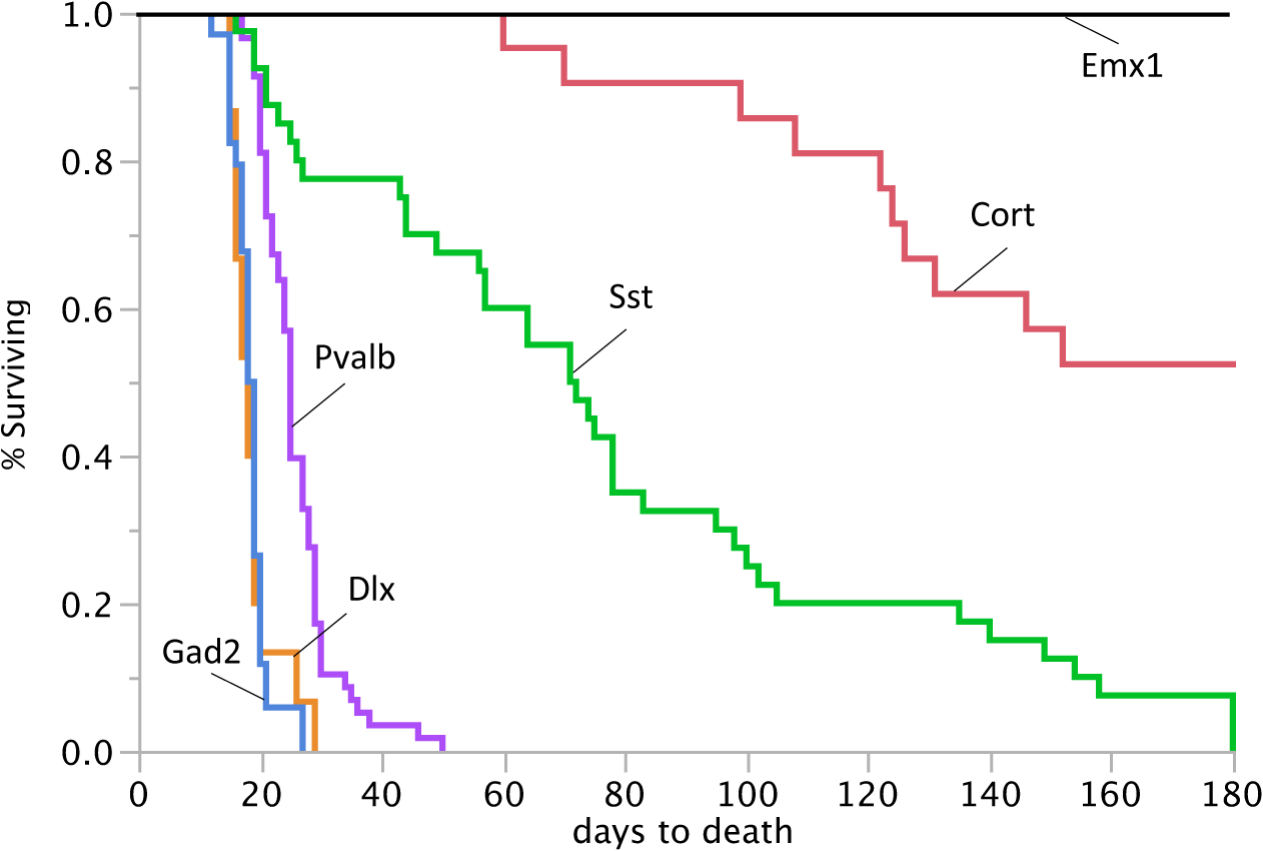
Reduced lifespan of *Dnm1* ^*Ftfl/flox*^ mice crossed to different Cre driver lines. Survival curves of *Dnm1* ^*Ftfl/flox*^ mice in conjunction with specific cre driver lines from postnatal day 0 until day 180. 100% of the *Gad2*-cre;*Dnm1* ^*Ftfl/flox*^ mice, 100% of the *Dlx5/6*-cre;*Dnm1* ^*Ftfl/flox*^ mice and 91% of the *Pvalb*-cre;*Dnm1* ^*Ftfl/flox*^ mice die before weaning. The *Emx1*-cre;*Dnm1* ^*Ftfl/flox*^ mice do not have a reduced lifespan.

*Dnm1* ^*Ftfl/+*^ and *Dnm1* ^*flox/flox*^ mice were crossed to *Gad2*-cre recombinase lines and then intercrossed to generate *Gad2*-cre;*Dnm1* ^*Ftfl/flox*^ compound heterozygous mice. *Gad2*-cre recombinase expresses in the majority of GABAergic inhibitory neurons [11]. The loss of *Dnm1* ^*Ftfl*^ heterozygosity in inhibitory neurons resulted in early onset lethal seizures with age at death ranging from postnatal 15–27 days old (P15-P27; Fig 4). Notably, the resulting mutants developed normally until two weeks of age and did not display any obvious neurosensory or locomotor deficits of the type that are always observed in *Dnm1* ^*Ftfl/Ftfl*^ homozygotes. These results demonstrate that selective GABAergic neuron deletion of wildtype *Dnm1* in the presence of the fitful allele is sufficient for seizure generation, but not the most overt locomotor comorbidities.

### Graded seizure onset and lethality corresponds to the range of *Dnm1* deletion

We reasoned that activating the EE genotype in a smaller subset of interneurons, such as the parvalbumin positive interneurons, would result in a less severe phenotype than activation in the majority of interneurons as above. Parvalbumin expressing interneurons represent about 40% of GABAergic interneurons and include fast spiking neurons that play a role in perisomatic inhibition of pyramidal neurons and cortical network oscillations [15–17]. Pyramidal cells are the primary excitatory neurons of the cortex and hippocampus. However, observations of seizure onset and lethality in the *Pvalb*-cre;*Dnm1* ^*Ftfl/flox*^ mice revealed a phenotype similar in severity to the *Gad2*-cre;*Dnm1* ^*Ftfl/flox*^ compound heterozygotes. The mice have early onset lethal seizures with a 91% fatality rate before P28 (Fig 4). We have video recorded these mice to confirm the seizure as the initiating event. The few mice that survived past 4 weeks of age succumbed to seizures within the next couple of weeks. In general, the young mice had no overt behavioral comorbidities. The few older surviving mice had visible locomotor dysfunction that appeared as a full body tremor or shakiness upon walking.

Somatostatin expressing (*Sst*) interneurons comprise about 30% of GABAergic neurons and have increased morphological and connective diversity than parvalbumin expressing neurons, but they also have a more discrete population [18,19]. In contrast to *Pvalb*-cre;*Dnm1* ^*Ftfl/flox*^ compound heterozygotes, *Sst*-cre;*Dnm1* ^*Ftfl/flox*^ mice have a variable seizure onset ranging from very early in life at P15 to later in life at P40 and older. Nevertheless, almost all of the mice have a lethal seizure event; 22% succumb before 4 weeks old and surviving mice ultimately have a lethal seizure before six months of age (Fig 4). Using the *Cort*-cre line which expresses in a limited amount of cortistatin positive interneurons [11], we see a longer lifespan for the mutants but 47% do succumb to seizures by six months of age (Fig 4). This suggests a graded seizure onset and lethality corresponding to the extent of *Dnm1* depletion and also points to network-specific effects that may contribute to decreased inhibition and the seizure disorder. We note that while this timing does overlap with the normal onset of seizures in germline *Dnm1* ^Ftfl/+^ heterozygotes, those seizures are never lethal.

### Diversity of EEG phenotypes from the Dnm1 defect

**Fig 5:**
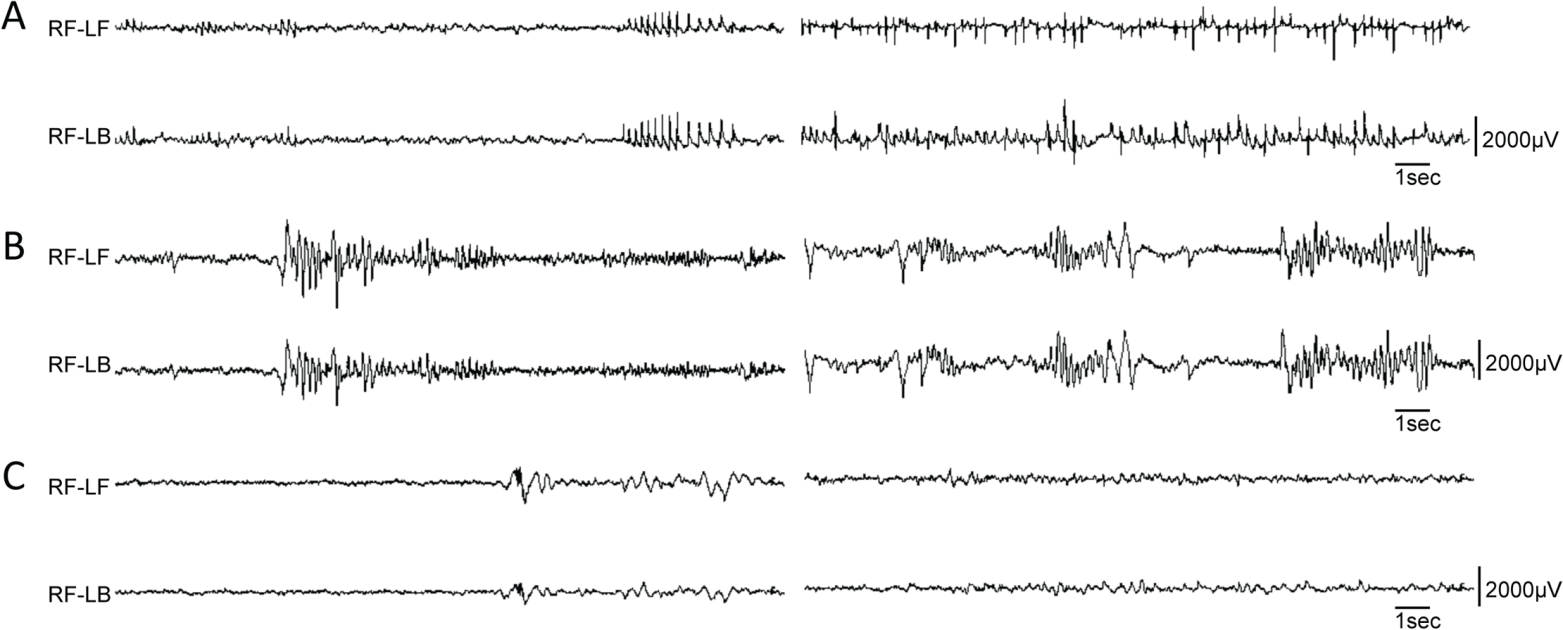
Variety of EEG seizures in different *Dnm1* ^*Ftfl*^ conditional mutants. Differential EEG traces from three different conditional models. (A) EEG analysis of one *Pvalb*-cre;*Dnm1* ^*Ftfl/flox*^ mouse that lived long enough for recording displayed remarkably frequent and synchronous spike-wave discharges when awake; two independent episodes are shown following each other, n = 1. (B) EEG analysis of a *Sst*-cre;*Dnm1* ^*Ftfl/flox*^ mouse shows high amplitude poly-spike and slow spike-waves that end abruptly, followed by a quiet baseline. This is suggestive of an aborted tonic-clonic seizure, of the type that when more severe or prolonged, is lethal. Shown are two independent examples of traces, n = 4. (C) EEG analysis of a *Emx1*-cre;*Dnm1* ^*Ftfl/flox*^ mouse showing normal EEG activity; two traces are shown. n = 2. RF, right-front; LF, left-front; LB, left-back.

We performed electroencephalography (EEG) analysis on the first *Pvalb-cre;Dnm1* ^*Ftfl/flox*^ mouse that survived beyond 3 weeks of age. EEG recordings from this mouse showed frequent, distinctive 5 Hz spike-wave discharges (SWDs) when the mouse was not moving (Fig 5A). It had on average 230 SWD episodes/hour of almost one second duration in general, with some extraordinary episodes of over 2 minutes. The antiepileptic drug, ethosuximide, completely blocked all SWD activity for a period of 15 minutes post injection. Of the other cre mice tested, only the *Sst-cre;Dnm1* ^*Ftfl/flox*^ mice showed any abnormal EEG activity including high amplitude poly-spike and slow spike-waves that end abruptly followed by a quiet baseline (Fig 5B). These data show the diversity of the EEG phenotypes arising from the dynamin 1 defect when spatial etiologies can be dissected systematically.

### Forebrain-specific activation of the *Dnm1* ^*Ftfl/null*^ EE phenotype

The lethality of the seizures suggests that the hindbrain/brainstem is involved. The brainstem region, and circuitry that initiates in the cortex and connects through the mid/hindbrain (including the cerebellum) to the brainstem, is important to regulate cardiovascular, respiratory and central nervous system autonomous functions. Malfunction of this system is suspected to underlie sudden unexpected death in epilepsy (SUDEP; [20]). To test the hypothesis that disruption of neurotransmission in the hindbrain/brainstem is sufficient for the lethality of the seizures, we used the forebrain GABAergic neuron specific cre recombinase *Dlx5/6*-cre [21] to remove *Dnm1* from a subset of forebrain inhibitory neurons and specifically leave the hindbrain intact. We expected the mice to have non-lethal seizures, but the *Dlx5/6*-cre;*Dnm1* ^*Ftfl/flox*^ mice experienced early onset lethal seizures, much like the *Gad2-Dnm1* ^*Ftfl/flox*^ mice (Fig 4). Before seizure onset mice appeared healthy, but 100% died by P16-P28. This suggests that acute deletion of *Dnm1* from the hindbrain is not required for lethality and that the seizure generates in the forebrain and most likely propagates through the rest of the brain and the brainstem. This may exemplify a primary defect that has secondary consequences.

### Activation of the *Dnm1* ^*Ftfl/null*^ EE phenotype in glutamatergic neurons is not sufficient for severe lethal seizures

**Fig 6:**
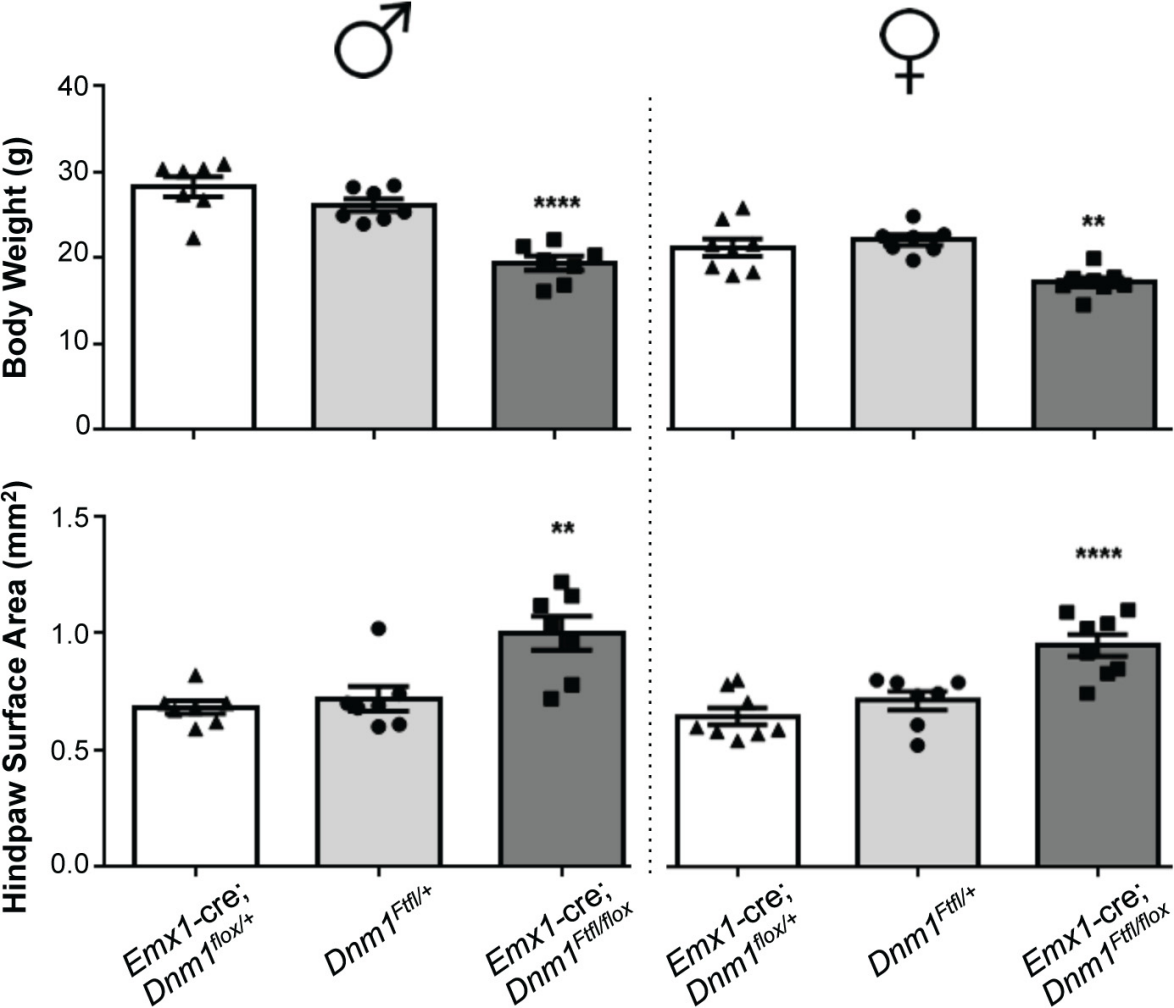
*Emx1*-cre; *Dnm1* ^*Ftfl/flox*^ mice are smaller than littermates and have postural differences. (Top) Adult mice were weighed at the onset of behavioral testing. Shown are male (left) and female (right) adult mice body weights. (Bottom) Dynamic weight bearing assay in freely moving animals measuring hindpaw surface area normalized to body weight. One-way ANOVA with Dunnett’s post hoc analysis (****P<0.0001, **P<0.01, v sex-matched WT control).

Deletion of wildtype *Dnm1* from cortical and hippocampal excitatory neurons with *Emx1*-cre [12] results in mice that are viable and fertile with normal lifespans (Fig 4). In contrast to GABAergic *Dnm1* compound heterozygotes (*Gad2*-cre*;Dnm1* ^*Ftfl/flox*^ or *Pvalb*-cre;*Dnm1* ^*Ftfl/flox*^ mice), these mice do not have early onset lethal seizures and also have normal EEG (Fig 5C). The mice do have later onset non-lethal seizures (68% of mice; n = 38) that begin between the ages of 53–146 days old. This is consistent with the fitful heterozygous phenotype and is expected due to the non-cre expressing neurons maintaining the heterozygous genotype (*Dnm1* ^*Ftfl/flox*^). Also, *Emx1*-cre;*Dnm1* ^*Ftfl/+*^ heterozygous littermate mice (without the floxed allele) have a similar seizure phenotype with onset at 57–182 days of age (70% of mice; n = 30). Initial observations suggested that the compound heterozygous mice (*Emx1*-cre*;Dnm1* ^*Ftfl/flox*^) exhibit several abnormalities which preceded seizure onset. We noted that they fail to thrive when weaned onto normal mouse chow solid diet (requiring a gel-based solid instead), are visibly smaller than littermates, have postural abnormalities, are hyperactive and demonstrate repetitive behavior in the form of stereotypical running around the perimeter of their homecage. Weights of adult mice show that the mutants are on average 20–30% smaller than control littermates (*Emx1*-cre;*Dnm1* ^*flox/+*^ vs *Emx1*-cre;*Dnm1* ^*Ftfl/flox*^; Fig 6, top). Analysis of posture using a dynamic weight bearing assay demonstrated that the mutants distribute more weight on their hindpaws than the forepaws relative to sex-matched controls (*Emx1*-cre;*Dnm1* ^*flox/+*^ vs *Emx1*-cre;*Dnm1* ^*Ftfl/flox*^; Fig 6, bottom) or to fitful heterozygous comparison mice (*Dnm1* ^*Ftfl/+*^ vs *Emx1*-cre;*Dnm1* ^*Ftfl/flox*^; Fig 6, bottom) confirming the altered posture observed. Neither the *Emx1*-cre;*Dnm1* ^*flox/+*^ nor *Emx1*-cre;*Dnm1* ^*Ftfl/+*^ littermates had these abnormalities. Table 3 summarizes the overall seizure and behavioral phenotypes observed in the six different cre lines documented above.

**Table 3:** Summary of the effects of each cre line in combination with the *Dnm1* ^*Ftfl/flox*^ genotype.

Cre line crossed to *Dnm1* ^*Ftfl/flox*^ mice	Seizure onset age (days old)	Seizure phenotype	EEG phenotype	Age at 100% lethality (days old)	Observed behavioral abnormalities
***Gad2*-cre**	15–27	lethal seizures	(N.A.)	27	none observed
***Pvalb*-cre**	19–50	lethal seizures	SWDs	50	tremor
***Sst*-cre**	15–40	lethal and non-lethal seizures	high amplitude poly-spikes	180	none observed
***Cort*-cre**	35–155	lethal and non-lethal seizures	(N.A.)	47% by P180	none observed
***Dlx5/6*-cre**	12–28	lethal seizures	(N.A.)	28	none observed
***Emx1*-cre**	53–146	non-lethal seizures	normal	(N.A.)	Small, hyperactive, altered posture, repetitive behaviors

### *Emx1*-cre;*Dnm1* ^*Ftf/flox*^ mice show abnormal locomotor, exploratory and repetitive behavior

To examine the neurobehavioral phenotypes observed and any potential subtle differences in the *Emx1*-cre;*Dnm1* ^*Ftf/flox*^ compound heterozygotes, mice were evaluated in a behavioral testing battery. We used littermate controls that carried the *Emx1*-cre and the *Dnm1* ^*flox*^ allele, but lacked the *Dnm1* ^*Ftfl*^ allele (*Emx1*-cre;*Dnm1* ^*flox/+*^) and also a comparison group that carried the *Dnm1* ^*Ftfl*^ allele, but not the *Emx1*-cre (*Dnm1* ^*Ftfl/+*^). This allowed us to compare the *Emx1*-cre compound heterozygotes to controls as well as fitful heterozygotes to ask if the behavioral deficits were due to off target effects of the cre, or due to the heterozygous fitful allele independent of neuron specific deletion of the wildtype allele. The mice were tested in a comprehensive battery and there were no significant differences observed for mutants compared to controls for several tests such as social approach, spontaneous alteration and grip strength. Furthermore, several test results were confounded by the hyperactivity demonstrated in the mutants (see below). The forced swim test, novel spatial recognition, rotarod and gait assessment were inconclusive due to the hyperactivity and/or repetitive behavior of the mutants.

**Fig 7:**
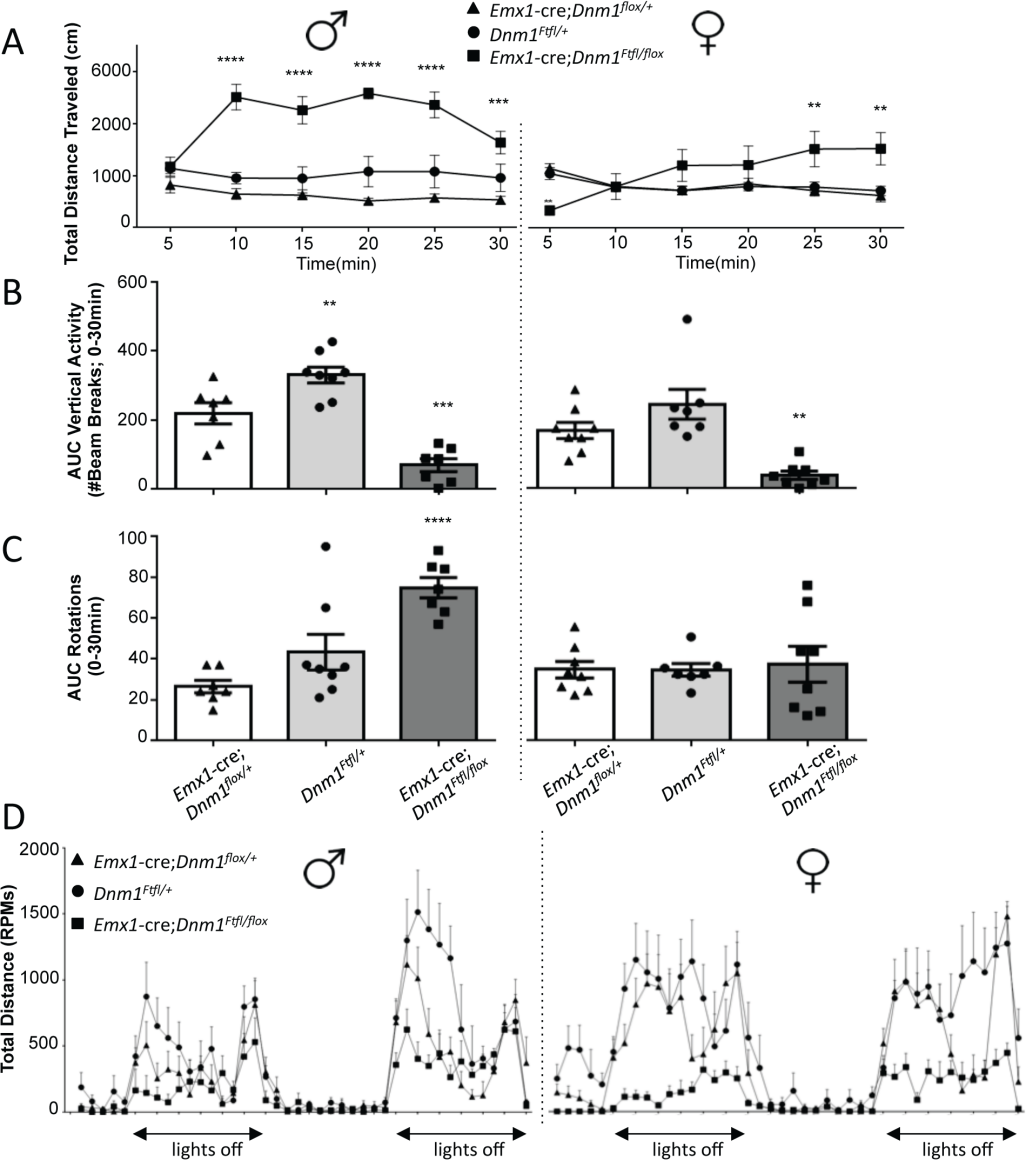
*Emx1*-cre;*Dnm1* ^*Ftfl/flox*^ mice demonstrate hyperactivity, stereotypical and altered activity. (A) Total distance traveled in the open field test, males on left, females on right. ****P<0.0001; two-way repeated measures ANOVA, Bonferroni post hoc analysis. (B) Vertical activity (rearing) in the open field test; males on left, females on right. One Way ANOVA with Dunnett’s post hoc test (***P<0.001, **P<0.01; vs WT control). (C) Repetitive behavior as measured by rotational behavior in the open field; males on left, females on right. One-way ANOVA (within sex) with Dunnett’s post-hoc test (****P<0.0001 vs WT control). (D) Activity measured in the wheel running assay. Total distance traveled (RPMs) was measured in 60 min time bins over a 48 hour period. Light/dark cycle is indicated below; males on left, females on right.

In the open field, male and female *Emx1*-cre*;Dnm1* ^*Ftfl/flox*^ mice demonstrated significant increases in total distance traveled relative to sex-matched control and fitful heterozygous mice confirming the observed hyperactivity (Fig 7A). Both male and female mutant mice also demonstrated significant reductions in rearing behavior, as measured by vertical beam breaks (Fig 7B). The reduced rearing may be confounded by, but is also consistent with, the increased hyperactivity and indicates altered exploratory activity. Interestingly, mutant males demonstrated a significant increase in stereotypical rotational behavior relative to control males, which was not observed in females (Fig 7C). This increase in rotational behavior is consistent with anecdotal observations of the repetitive running behavior observed in the homecage and confirms the repetitive behavior of the mutants. Male and female mutants also demonstrated significant disruptions in wheel running activity, both total time spent running and speed, over a 48-hour period relative to sex-matched controls (Fig 7D). Mice were also evaluated for cognitive phenotypes, but all tasks except for one were confounded by the hyperactivity observed in the mutants. The spontaneous alternation task was successfully carried out and demonstrated intact hippocampal working memory in the mutants compared to controls and fitful heterozygous mice (S2 Fig).

### Altered anxiety and depressive related behaviors in *Emx1*-cre;*Dnm1* ^*Ftfl/flox*^ mice

**Fig 8:**
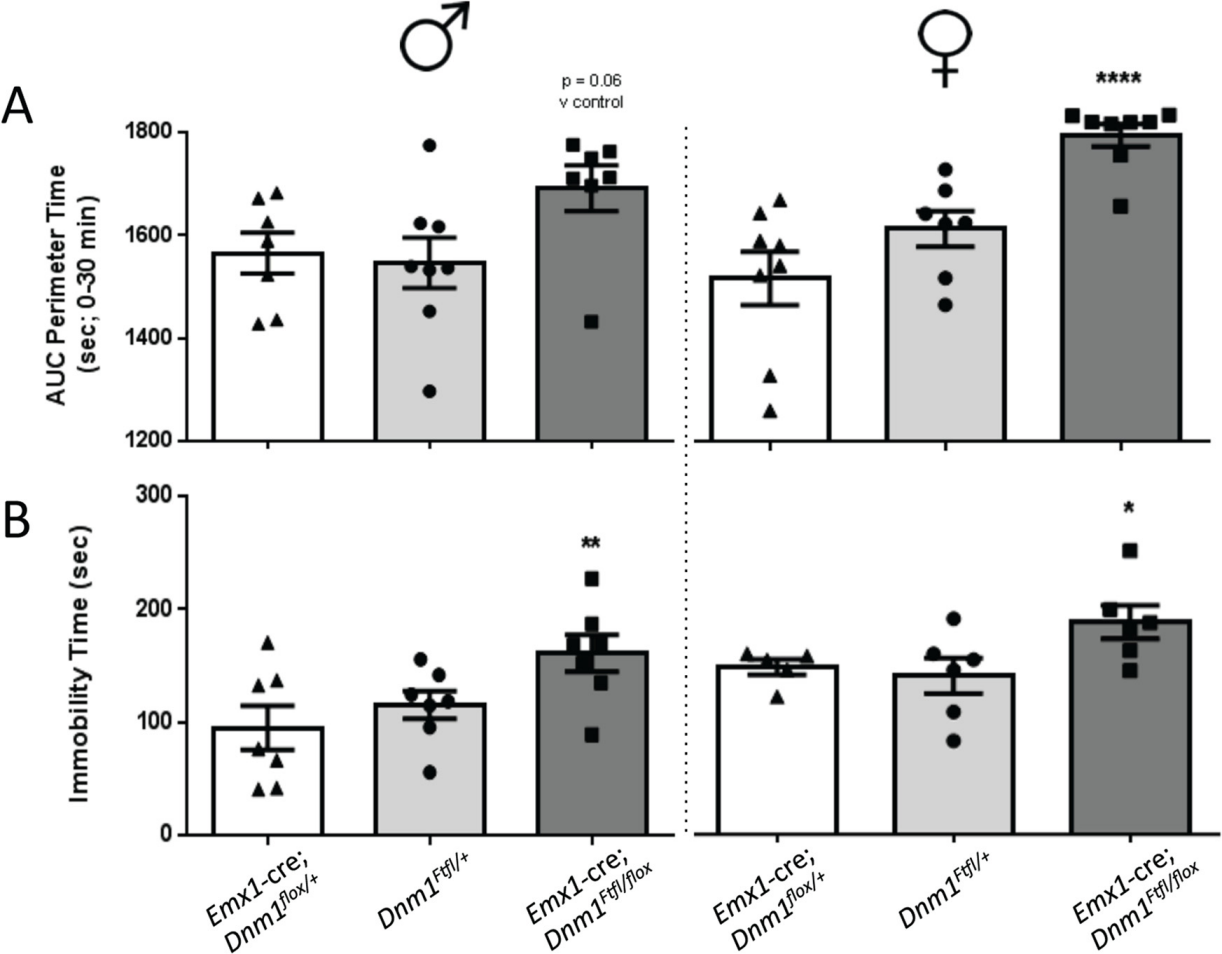
Anxiety- and depression-related behaviors of *Emx1*-cre;*Dnm1* ^*Ftfl/flox*^ mice. (A) Amount of time spent at the perimeter was measured in the open field for 30 min; males on left, females on right. (B) Immobility time (sec) was measured in the tail suspension test; males on left, females on right. One-way ANOVA (within sex) with Dunnett’s post hoc analysis (****P<0.0001, **P<0.01, *P<0.05; v WT control).

*Emx1*-cre compound heterozygotes exhibited increases in time spent at the perimeter of the open field relative to sex-matched controls which is indicative of an anxiogenic-like phenotype (Fig 8A). Male and female mutant mice also demonstrated significant increases in immobility times relative to sex-matched controls in the tail suspension test (Fig 8B), indicative of a depressive-like behavior.

### Altered dopaminergic and glutamatergic response in *Emx1*-cre;*Dnm1* ^*Ftfl/flox*^ mice

**Fig 9:**
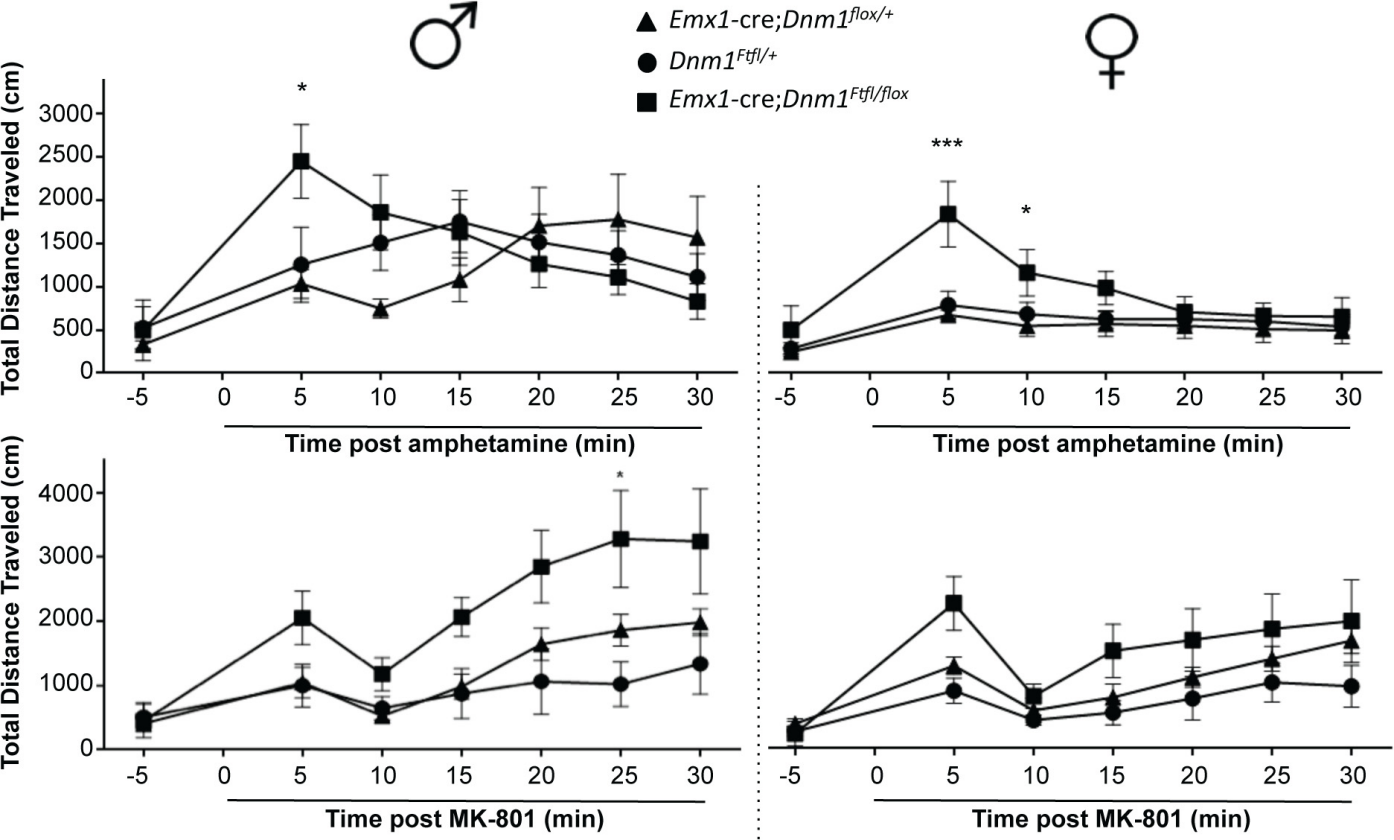
Altered dopaminergic and glutamatergic response. Hyperactivity induced by amphetamine (top graphs; males left, females right) or by challenge with MK-801 (bottom graphs; males left, females right) as measured by total distance traveled in the open field after administration of drug. Two-way repeated measures ANOVA (time x same sex genotype) with Bonferroni post-hoc comparisons (***P<0.001, *P<0.05).

Stimulation of locomotor activity by amphetamine was exacerbated in both male and female mutant *Emx1*-cre;*Dnm1* ^*Ftfl/flox*^ mice relative to controls immediately post drug administration (Fig 9, top). Challenge with the NMDA antagonist MK801 also resulted in an exacerbated increase in locomotor activity in mutant males compared to male controls, while there was no significant difference in female mice across genotypes (Fig 9, bottom).

## Discussion

The large GTPase *DNM1* has recently been confirmed as an EE gene. DNM1 patients have seizures that begin early in life and usually manifest as infantile spasms. These patients exhibit developmental, cognitive, behavioral and neurological deficits that do not appear to respond to traditional therapy.

Seizure onset in DNM1 patients ranges from 2–13 months of age and usually presents with infantile spasms. The seizure type manifests in various forms as the patient ages, ranging from absence seizures to generalized tonic clonic seizures. EEG recordings in these patients reveal varied epileptiform discharges initiating as hypsarrhythmia, and evolving from slow generalized spike-wave discharges to paroxysmal fast activity [1,3]. Patients have not responded well to antiepileptic therapy, with the majority being resistant. Interestingly, two patients, with DNM1 middle domain mutations, have had seizure control on the ketogenic diet while two other patients, with GTPase domain mutations, had no relief on the diet [1,3]. Commonly, the patients were reported to have hypotonia with one child presenting with ataxia and mild tremor. All patients have severe intellectual disability and developmental delay. One child is reported to have autism spectrum disorder [3]. For two patients, evidence of developmental delay was noted before the onset of epilepsy suggesting an effect of DNM1 variants on neurodevelopment unrelated to seizure activity [3].

Fitful mice recapitulate the disorder documented in DNM1 patients and exhibit an early onset seizure syndrome and comorbid behavioral alterations. Recently, Dhindsa *et al* [4] characterized several human DNM1 variants at the cellular level. Noticeably, the human G359A variant, located in the same domain as fitful (middle; see Fig 1), had a cellular phenotype consistent with fitful when expressed in Cos-7 cells. Both mutations conferred a decrease in endocytosis, a reticular dynamin 1 distribution and inefficient oligomerization [4,5]. The other human variants studied (A177P, K206N; Fig 1), located in the GTPase domain and expected to disrupt GTPase activity, had decreased endocytosis, altered localization and vesicle abnormalities when overexpressed in cells [4]. While some of the human variants and fitful reside in different domains of dynamin 1, they all significantly decrease endocytosis suggestive of a dominant-negative effect. It is plausible that the GTPase domain and middle domain mutations cause dynamin 1 protein dysfunction by different mechanisms (GTPase activity versus oligomerization) that ultimately result in defective dynamin 1 activity and inefficient endocytosis. In the case of the patients, the dominant negative effect of DNM1 results in a generally similar overall phenotype suggesting a similar pathway, the most probable mechanism being decreased or inefficient endocytosis resulting in synaptic vesicle pool depletion. Inhibitory neurons are more sensitive to a lack of *Dnm1* possibly due to their tonic activity [22] and lack of vesicle replenishment in these neurons would cause decreased inhibition and network hyperactivity.

Many brain disorders, including the epileptic encephalopathies as well as Fragile X syndrome, Rett syndrome, tuberous sclerosis, Lennox-Gastaut syndrome and West syndrome (reviewed in [23,24]), manifest early in life and are accompanied by spasms and/or seizures that are usually intractable. The high level of cognitive and behavioral anomalies that co-occur in these patients with syndromes of varying genetic etiologies are suggestive of disruptions during brain development. Patients exhibit developmental, cognitive, behavior and neurological deficits that often do not respond to therapies and may lead to early death. The relentless seizure activity is correlated with, and generally assumed to contribute to, the progressive cognitive decline. This may not be the case at least for DNM1 encephalopathy, suggested by the fact that two of the DNM1 patients became seizure free on the ketogenic diet but still have the above mentioned syndromic features and developmental delay was noted in two patients even before the onset of epilepsy. Using the fitful mice as a model of the severe DNM1 EE, we have demonstrated clear separation of the seizure etiology from several major behavioral and neurological comorbidities. This strongly supports the conclusion that amelioration of the seizures is unlikely to curtail the severe behavioral deficits.

Wildtype *Dnm1* deletion from the majority of *Dnm1* ^*Ftfl*^ inhibitory neurons is sufficient to cause early onset epilepsy and premature death, without other obvious behavioral impairment. The seizure etiology varies depending on the amount of neurons that have deleted wildtype dynamin 1, but also on the specific type of neuron or circuitry. Deleting wildtype *Dnm1* from the majority of interneurons results in 100% loss of viability before weaning age in inhibitory specific compound heterozygotes. Sparse interneuron deletion results in occasional adult onset seizures that are rarely lethal as observed with *Cort*-cre. Interestingly, parvalbumin expressing interneurons which account for about 40% of GABAergic neurons appear to have a very important role in controlling excitation as deletion of wildtype *Dnm1* from these neurons has very severe consequences. Comparatively, the compound heterozygous fitful mutation expressed in somatostatin positive interneurons (about 30% of GABAergic neurons) leads to a less severe disorder. Notably, we observed differences in EEG recordings- indicating alterations in seizure pathology- from *Dnm1* ^*Ftfl/flox*^ mice crossed to the *Pvalb* and the *Sst* cre driver lines. These suggest that there may be network-specific effects influencing the magnitude and scope of discrete components of the seizure disorder. This type of circuitry malfunction, while beyond the scope of this study, will be critical to dissect in the future.

Conversely, impaired synaptic function due to *Dnm1* mutation in excitatory neurons results in significant behavioral impairment prior to the emergence of seizures. This suggests that at least several major behavioral comorbidities are not explicitly caused by the seizures, thus separating their etiology. These mice are visibly smaller, hyperactive, repetitive and have reduced rearing (exploratory) activity in their cages. Interestingly, hyperactivity and lower exploratory activity have been reported for autism spectrum disorder (ASD) patients [25] and repetitiveness is a core characteristic [26,27]. The excitatory *Dnm1* deleted mice also displayed decreased motivation, increased depression and anxiousness. Due to the hyperactivity in the mutants, it precluded the ability to evaluate their cognitive phenotype without ruling out locomotor confounds in tests of learning and memory with the exception of spontaneous alternation task which demonstrated intact hippocampal working memory (see supplemental data). Taken together, the observations suggest that presynaptic dysfunction of the excitatory neurons leads to behavioral alterations associated with psychiatric comorbidities, such as documented for ASD and hyper-reactivity to glutamatergic and dopaminergic blockade.

It seems likely that there could also be behavioral abnormalities associated with the GABAergic neuron phenotypes, perhaps even ones that appear to be normal in the excitatory compound heterozygotes. However, the challenge is that the very severely affected mice do not live long enough to begin behavioral assays with them. An option for the future will be to analyze the behavior of the GABAergic compound heterozygotes that live longer using the inhibitory cres with graded expression, such as the *Sst*-cre or *Cort*-cre.

There are limitations to the genetic system utilized in this study. The most obvious limitation being the difficulty in quantitating the actual extent and specificity of deletion of the floxed allele. We have addressed this by analyzing the *Emx1*-cre;*Dnm1* ^*Ftfl/flox*^ line reasoning that this cre expresses in the majority of glutamatergic cells in the brain and we should be able to observed a change in Dnm1 levels in such a large population of cells. Indeed, we do see a 50% decrease in Dnm1 levels in these mice, indicating a deletion of one *Dnm1* allele. Further, as a correlate, we observe a graded seizure phenotype in the mice that corresponds to the amount of inhibitory neurons that we are targeting for deletion. While indirect, this observation supports the concept that the floxed allele is deleting in a predicted proportion of the neurons.

Another potential caveat regards the degree to which one can interpret the clinical significance of genetically manipulating a subset of neurons in a model system of a human disorder where the defect presumably occurs in all neurons that express the gene. For example, in some mouse models such as the Dravet Syndrome SCN1a mutants, disrupting the gene in individual populations of neurons does not replicate the human phenotype, suggesting that an intact inhibitory/excitatory circuit is required for the full disorder [28]. Although the small number of reported *DNM1* patients have a uniformly severe disorder, we do not know the degree of *de novo* variant mosaicism in these patients. It is difficult to know whether even partial DNM1 genotypes can also lead to severe outcomes or whether mosaic outcomes would be milder as has been observed in some developmental excitability disorders, such as X-linked Rett Syndrome [29]. In our case, to varying degrees all inhibitory neuron cre mice displayed the lethal seizure phenotypes including sparser *Sst*- and *Cort*-cre, demonstrating that widespread dynamin-1 dysfunction is not required for severe seizures at least. Regardless, while we cannot know formally that severe seizures observed in *Dnm1* ^Ftfl/flox^ inhibitory cre conditional mutants have the same network pathophysiology as those that occur in the germline condition, such uncertainty does not affect our key observation that overtly impaired excitatory conditional mutants do not have a severe seizure disorder of any kind.

Wildtype *Dnm1* deletion from *Dnm1* ^*Ftfl*^ inhibitory neurons is sufficient to cause early onset epilepsy and premature death, without other obvious behavioral impairment. Wildtype *Dnm1* deletion from *Dnm1* ^*Ftfl*^ excitatory neurons results in behavioral impairment without seizures, strongly *suggesting that the behavioral comorbidities are not caused by the seizures in the germline mutant mice*, *with clear implications for human DNM1 patients*. Together these results suggest that preventing seizures *per se* in *Dnm1* fitful mice, and potentially EE patients, may not prevent all of the severe comorbidities.

## Materials and Methods

### Generation and maintenance of mouse lines

All mice were housed and procedures performed with approval of Institutional Animal Care and Use Committee (IACUC). All mice were obtained from The Jackson Laboratory, maintained in a room with a 14 hour light on/10 hour light off cycle, and given free access to LabChow meal and water.

The fitful mice, B6.SZ, arose at The Jackson Laboratory (Bar Harbor, ME) as a spontaneous mutation on the C57BL/6J inbred strain in 2000 [5]. *Dnm1* ^*tm2*.*1Pdc/J*^ mice contain *loxP* sites flanking exons 2–4 of the *Dnm1* gene (Fig 2; [7]). Mice obtained from The Jackson Laboratory were backcrossed and maintained on a C57BL/6J background. We refer to mice with this allele as the floxed mice, or *Dnm1* ^*flox/flox*^. Homozygous floxed mice are viable, fertile and normal in size with no obvious phenotypic abnormalities. Mouse lines obtained from JAX: STOCK *Gad2* ^*tm2(cre)Zjh*^/J (backcrossed to C57BL/J 6 generations), B6;129P2-*Pvalb* ^*tm1(cre)Arbr*^/J, STOCK *Sst* ^*tm2*.*1(cre)Zjh*^/J (backcrossed to C57BL/J 6 generations), STOCK *Cort* ^*tm1(cre)Zjh*^/J (backcrossed to C57BL/J 5 generations), STOCK Tg(*Dlx6a*-cre)1^Mekk^/J (backcrossed fully to C57BL/J), B6.129S2-*Emx1* ^*tm1(cre)Krj*^/J (backcrossed fully to C57BL/6), B6.Cg-*Gt(ROSA)26Sor* ^*tm9(CAG-tdTomato)Hze*^/J.

### Genotyping

Genotypes were determined by PCR using tail DNA. Genotyping *Dnm1* ^*Ftfl*^ (fitful) was done as previously [5]. The *Dnm1* ^*tm2*.*1Pdc/J*^ allele was PCR amplified with specific primers (*condF*, 5’CAGCTGGGTATAATGAGGCCTCATC3’; *condR*, 5’GCATGGGTGCAC TCACATACACAA3’). Presence of *cre* in the various strains was determined by PCR amplification with generic cre primers (*oIMR1084*, 5’GCGGTC TGGCAGTAAAAACTA C3’; *oIMR1085*, 5’GTGAAACAGCATTGCTGTCACTT3’; *oIMR7338*, 5’CTAGGCCACAGAATTGAAAGATCT3’; *oIMR7339*, 5’GTAGGTGGAAAT TCTAGCATCATCC3’).

### Immunohistochemistry

Mice were anesthetized with tribromoethanol, perfused with 0.1M PBS then 4% paraformaldehyde in 0.1M PBS and postfixed overnight. Sagittal sections of 60–70 μm thick were cut on a vibratome. Sections were blocked and permeabilized for 4.5 hours in PBS 1% BSA 4% Goat serum and 2.3% Triton-X, then incubated in primary antibody (see dilutions below) overnight at 4 degrees Celsius while rocking. Sections were then washed and incubated in 1 hour in secondary antibody (goat anti-mouse conjugated to AlexaFluor 488; Invitrogen), 1:1000 in PBS 1% BSA 4% Goat serum and 0.3% Triton-X. After another wash, sections were stained for 5 minutes with DAPI (1:1000 in PBS), washed again, and mounted on Shandon Colorfrost Plus slides (Thermo Scientific) with Fluorogel (Electron Microscopy Sciences). Confocal images were taken on a Leica SP5 microscope.

### Reverse-transcription PCR

Total RNA was prepared from brains with Trizol (Invitrogen) following the manufacturer's suggested conditions and protocol. RNA (0.5μg) was reverse transcribed with AMV reverse transcriptase (Promega). cDNA was diluted and amplified for 25 cycles at an annealing temperature of 55°C with primers spanning Dnm1 exons 9 to 12 (*Dnm1ex9F*, 5′-GAACTGCGA AGGGAGATCAG-3′; *Dnm1ex12R*, 5′-GGTCACAATTCGCTCCATCT-3′).

### Western blot

Protein extracts were made in IP lysis buffer (10 mM HEPES pH 7.5, 137 mM NaCl, 0.4% NP-40, 10% glycerol) with Complete-mini proteinase inhibitor mix (Roche) added fresh. Extracts were quantified using the Bradford reagent (Bio-Rad). Extracts (50 μg protein) were diluted in Laemmli buffer, incubated at 95°C for 5 minutes, resolved by SDS-PAGE and transferred to nitrocellulose membrane. All membrane blotting steps were carried out in TBS plus Tween (TBST). Blots were blocked in 5% milk, then incubated at RT with primary antibody (for specific dilutions see below) for 4 hours, HRP-conjugated secondary antibody (1:15000) for 1h and visualized with Luminata Forte (Millipore). Membranes were incubated with Restore Western blot stripping buffer (Thermo Scientific) at room temperature for 10 minutes while shaking to remove antibodies for subsequent hybridization. Primary antibodies used were dynamin-1 (1:3000; Thermo Scientific, PA1-660), actin (1:1000; Abcam, ab3280). Secondary antibodies used were HRP anti-mouse (Thermo Scientific), HRP anti-rabbit (BioRad).

### Spontaneous seizure analysis and 24-hour video recording

Mice were observed for age-of-onset, frequency and severity in their homecage for handling evoked seizures during weekly cage changing for at least 175 days. 24-hour recording was done using infrared-video on isolated mice in a chamber.

### EEG

Adult mice aged between 6 and 8 weeks were anesthetized with tribromoethanol (400 mg/kg i.p.). Small burr holes were drilled (1 mm anterior to the bregma and 2 mm posterior to the bregma) on both sides of the skull 2 mm lateral to the midline. Four teflon-coated silver wires were soldered onto the pins of a microconnector (Mouser electronics, Texas). The wires were placed between the dura and the brain and a dental cap was then applied. The mice were given a post-operative analgesic of carprofen (5 mg/kg subcutaneous) and allowed a minimum 48 h recovery period before recordings. Differential amplification recordings were recorded between all four electrode pairs, providing 6 channels for each subject. Mice were connected to the EEG Stellate Lamont Pro-36 programmable amplifier (Lamont Medical Instruments, Madison, WI) for a 24-hour period with accompanying video recording. EEG data were recorded with Stellate Harmonie software (Stellate Systems, Inc., Montreal, Canada). The antiepileptic drug, ethosuximide (Sigma), was dissolved in saline and administered by intraperitoneal injection at a dose of 200mg/kg. EEG and video recordings were continued for a further hour.

### Behavioral testing

Adult male and female *Emx1*-cre;*Dnm1* ^*flox/+*^ (control), *Dnm1* ^*Ftfl/+*^ (fitful heterozygotes) and *Emx1*-cre;*Dnm1* ^*Ftfl/flox*^ (mutant) mice (n = 7–8 per sex, per genotype) were evaluated in a battery of behavioral tests. Order of testing was as follows: Open field, tail suspension, dynamic weight bearing, wheel running, amphetamine stimulation, MK-801 stimulation. Behavioral tests were conducted by trained technicians blind to genotype. All testing was performed during the light cycle with at minimum 1 day of rest between tests and 1 week between pharmacological challenges. Sexes were independently analyzed due to the sexually dimorphic behaviors. A one-way ANOVA within sex followed by a Dunnett’s post-hoc test with wildtype as control was utilized to compare data for tests defined by one factor. A two-way repeated measures ANOVA (treatment x time) with a Bonferroni post-hoc analysis was computed for tests involving more than one treatment and multiple timepoints/trials. Explicit use of these measurements is stated in the figure legends.

#### Spontaneous open field activity

Mice were acclimated to an anteroom adjacent to the testing room for a minimum of 60 min. Subjects were individually placed into open field arenas (40 x 40 x 40 cm; Omnitech Electronics, Inc, Columbus, OH USA) housed in sound attenuated chambers with chamber lighting ~ 500 lux. Distance traveled (cm), vertical activity, perimeter and center time (sec) and rotational behavior were recorded via infrared beams (Versamax Software; Omnitech Electronics, Inc.) over the course of a 30 min period.

#### Tail suspension test

Mice were acclimated to the testing room for a 60 min period under ambient lighting conditions (~ 20 lux). During the test, subjects are suspended by their tails with laboratory tape to a flat metal bar connected to a force transducer (Med-Associates, St. Albans, VT USA) for a 6 minute testing period. Immobility time is automatically calculated by Med Associates software. A significant number of female *Emx1-cre;Dnm1* ^*flox/+*^ mice (N = 3 of 8), one *Dnm1* ^*Ftfl/+*^ female (N = 1 of 8) and one *Emx1-cre;Dnm1* ^*Ftfl/flox*^ female (N = 1 of 8) were excluded from the assay due to tail climbing which is common in C57Bl/6J mice in this assay [30].

#### Dynamic weight bearing

Weight bearing and postural abnormalities were evaluated in the Dynamic Weight Bearing Apparatus (EB-Instruments, Pinellas Park, FL USA). Subjects were weighed and acclimated to the testing room for a 60 min habituation period. Following acclimation, mice were individually placed in the chamber and allowed to move freely within the apparatus for a 3–5 min test periodDuring the data capture, the raw data for each paw is synchronized with the images from a video camera and the averaged values are recorded with a sampling rate of 10Hz. Paw surface area (mm^2^) was normalized to body weight.

#### Wheel running

Mice were individual housed in a new cage with ad libitum food and water, and a running wheel equipped with a wireless transponder (Med-Associates, St. Albans, VT USA). Mice were left undisturbed throughout the 48 hour recording period. Data were analyzed for distance traveled (revolutions per minute; rpm) and time spent running (min).

#### Amphetamine and MK-801 stimulated locomotor activity

Mice were habituated to the open field arenas for a 90 min session under ambient lighting (~50 lux). The arenas used for this test were the same open field arenas previously used to evaluate spontaneous activity (as described above). At the conclusion of the 90 min session, mice were challenged with amphetamine (1 mg/kg, i.p.), immediately returned to the chamber, and behavior was recorded for an additional 60 min period. One week following amphetamine challenge, mice were subjected to an acute challenge with the NMDA antagonist MK-801 (0.178 mg/kg, sc) following identical procedures used for the amphetamine challenge. Test compounds: d-amphetamine hemisulfate and (+) MK-801 hydrogen maleate were purchased from Sigma (St. Louis, MO USA) and dissolved fresh in NaCl. All drug concentrations were corrected for % active moiety of the free base and administered at an injection volume of 10 ml/kg.

## Supporting Information

S1 Fig Reduced levels of Dnm1 in *Emx1*-cre;*Dnm1* ^*Ftfl/flox*^ mice. (A) Representative western blot showing reduced levels of Dnm1 protein relative to loading control actin levels in lysates from cortex. All animals expressed *Emx1*-cre with various *Dnm1* genotypes labeled above lanes. Mice carrying a floxed allele have a decrease in Dnm1 protein. (B) Quantification of Dnm1 protein levels. N = 3.
(TIF)

S2 Fig Intact hippocampal memory in *Emx1*-cre;*Dnm1* ^*Ftfl/flox*^ mice. Quantification of the number of entries into each of 3 different arms of the Y maze in sequence. % alternation is depicted.
(TIF)
