# Supplementary figures and images for: Independent Neuronal Origin of Seizures and Behavioral Comorbidities in an Animal Model of a Severe Childhood Genetic Epileptic Encephalopathy
Source: PLoS Genet. 2015 Jun 30;11(6):e1005347. doi: 10.1371/journal.pgen.1005347 (PMC4488318; doi:10.1371/journal.pgen.1005347)

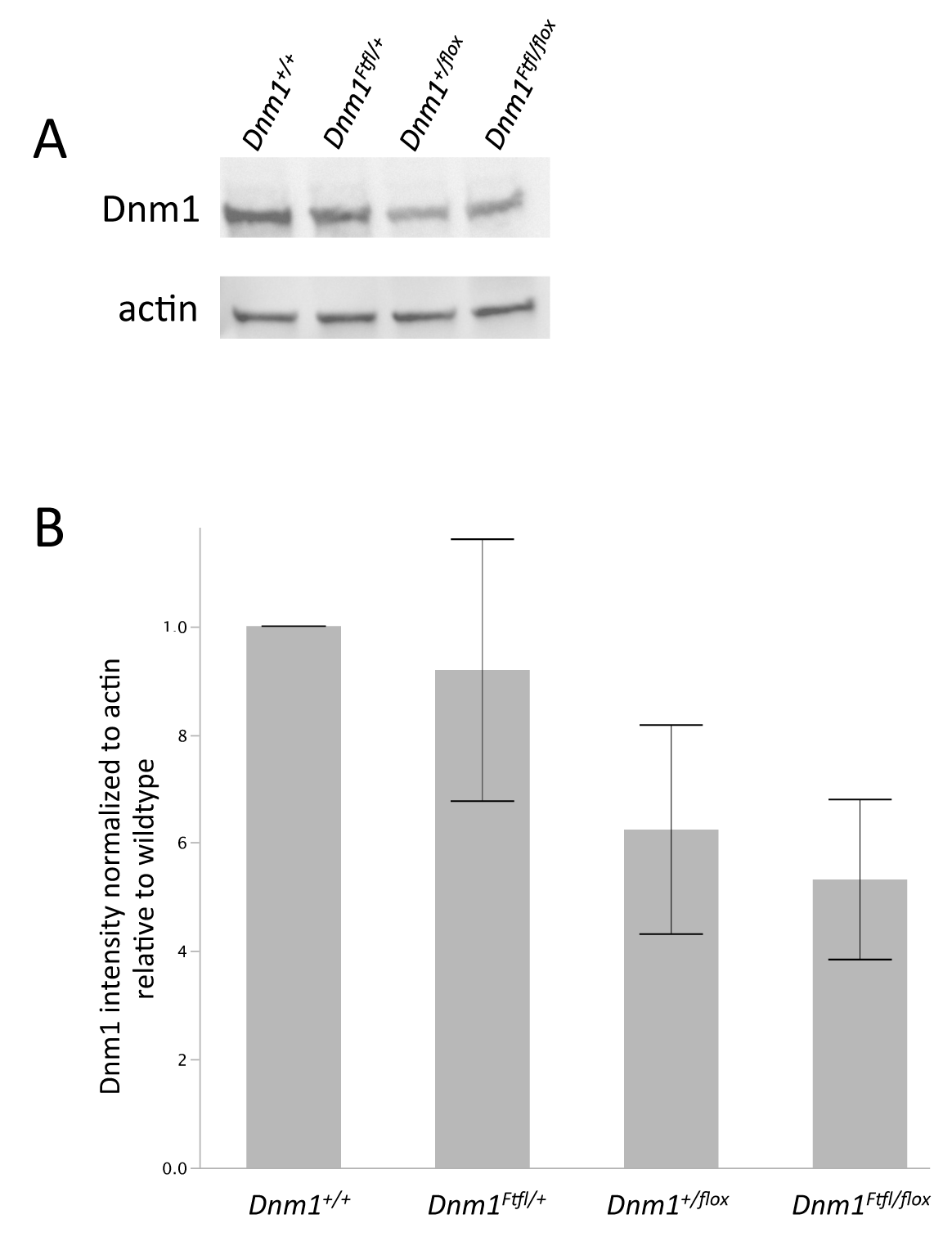

Supplement: S1 Fig — (A) Representative western blot showing reduced levels of Dnm1 protein relative to loading control actin levels in lysates from cortex. All animals expressed Emx1-cre with various Dnm1 genotypes labeled above lanes. Mice carrying a floxed allele have a decrease in Dnm1 protein. (B) Quantification of Dnm1 protein levels. N = 3. (TIF) [file pgen.1005347.s001.tif]

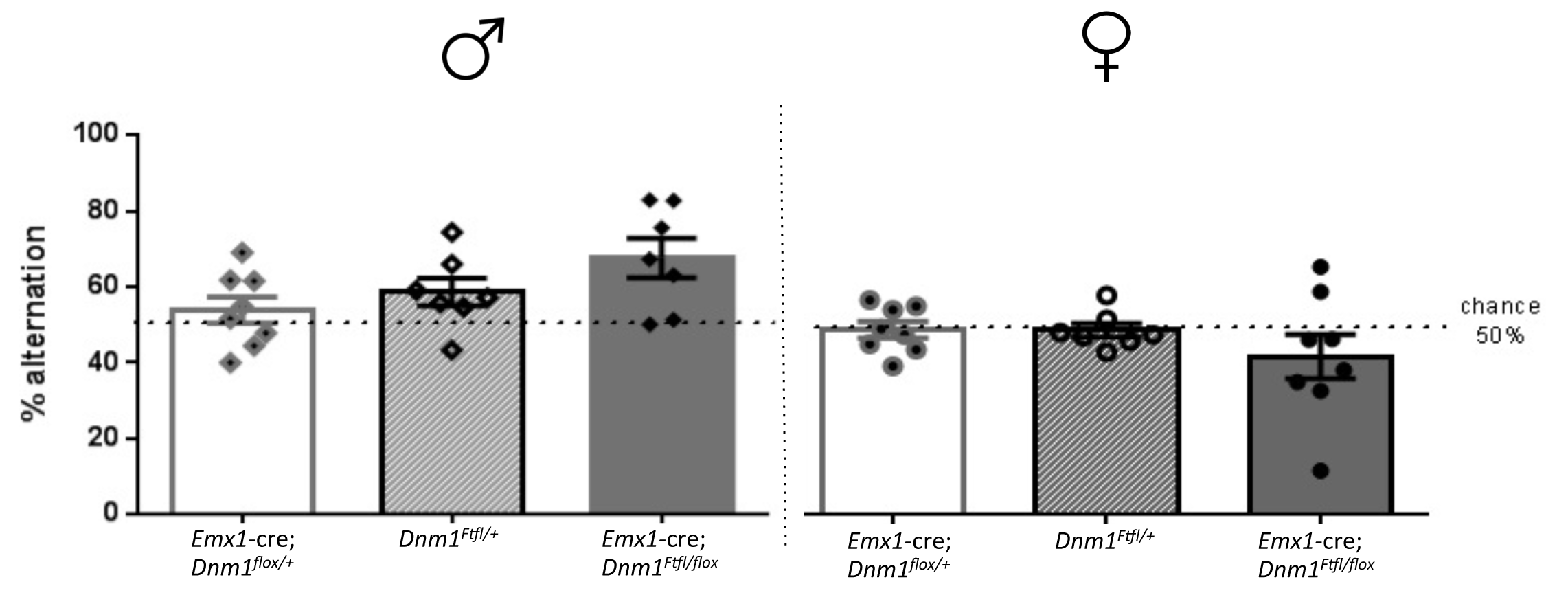

Supplement: S2 Fig — Quantification of the number of entries into each of 3 different arms of the Y maze in sequence. % alternation is depicted. (TIF) [file pgen.1005347.s002.tif]
